# Low-Dose Ionizing Radiation Modulates Microglia Phenotypes in the Models of Alzheimer’s Disease

**DOI:** 10.3390/ijms21124532

**Published:** 2020-06-25

**Authors:** Sujin Kim, Hyunju Chung, Han Ngoc Mai, Yunkwon Nam, Soo Jung Shin, Yong Ho Park, Mi Joo Chung, Jong Kil Lee, Hak Young Rhee, Geon-Ho Jahng, Youngkyong Kim, Yu Jin Lim, Moonkyoo Kong, Minho Moon, Weon Kuu Chung

**Affiliations:** 1 Department of Biochemistry, College of Medicine, Konyang University, 158, Gwanjeodong-ro, Seo-gu, Daejeon 35365, Korea; aktnfl3371@naver.com (S.K.); yunkwonnam@gmail.com (Y.N.); tlstnzz83@gmail.com (S.J.S.); znf900809@naver.com (Y.H.P.); 2 Department of Core Research Laboratory, Medical Science Research Institute, Kyung Hee University Hospital at Gangdong, Seoul 05278, Korea; hjchung@khu.ac.kr; 3 Department of Radiation Oncology, Kyung Hee University Hospital at Gangdong, Seoul 05278, Korea; maingochan244@gmail.com (H.N.M.); mjwithu@khnmc.or.kr (M.J.C); 4 Department of Pharmacy, College of Pharmacy, Kyung Hee University, Seoul 02447, Korea; jklee3984@gmail.com; 5 Department of Neurology, Kyung Hee University Hospital at Gangdong, Seoul 05278, Korea; azzo73@gmail.com; 6 Department of Radiology, Kyung Hee University Hospital at Gangdong, Seoul 05278, Korea; ghjahng@gmail.com; 7 Department of Radiation Oncology, Kyung Hee University Medical Center, Kyung Hee University School of Medicine, Seoul 02447, Korea; icarus070@hanmail.net (Y.K.); yujindw@naver.com (Y.J.L.); kongmoonkyoo@khu.ac.kr (M.K.)

**Keywords:** low-dose ionizing radiation, microglia, amyloid-beta, Alzheimer’s disease, TREM2, M1/M2

## Abstract

Alzheimer’s disease (AD) is the most common type of dementia. AD involves major pathologies such as amyloid-β (Aβ) plaques and neurofibrillary tangles in the brain. During the progression of AD, microglia can be polarized from anti-inflammatory M2 to pro-inflammatory M1 phenotype. The activation of triggering receptor expressed on myeloid cells 2 (TREM2) may result in microglia phenotype switching from M1 to M2, which finally attenuated Aβ deposition and memory loss in AD. Low-dose ionizing radiation (LDIR) is known to ameliorate Aβ pathology and cognitive deficits in AD; however, the therapeutic mechanisms of LDIR against AD-related pathology have been little studied. First, we reconfirm that LDIR (two Gy per fraction for five times)-treated six-month 5XFAD mice exhibited (1) the reduction of Aβ deposition, as reflected by thioflavins S staining, and (2) the improvement of cognitive deficits, as revealed by Morris water maze test, compared to sham-exposed 5XFAD mice. To elucidate the mechanisms of LDIR-induced inhibition of Aβ accumulation and memory loss in AD, we examined whether LDIR regulates the microglial phenotype through the examination of levels of M1 and M2 cytokines in 5XFAD mice. In addition, we investigated the direct effects of LDIR on lipopolysaccharide (LPS)-induced production and secretion of M1/M2 cytokines in the BV-2 microglial cells. In the LPS- and LDIR-treated BV-2 cells, the M2 phenotypic marker CD206 was significantly increased, compared with LPS- and sham-treated BV-2 cells. Finally, the effect of LDIR on M2 polarization was confirmed by detection of increased expression of TREM2 in LPS-induced BV2 cells. These results suggest that LDIR directly induced phenotype switching from M1 to M2 in the brain with AD. Taken together, our results indicated that LDIR modulates LPS- and Aβ-induced neuroinflammation by promoting M2 polarization via TREM2 expression, and has beneficial effects in the AD-related pathology such as Aβ deposition and memory loss.

## 1. Introduction

Alzheimer’s disease (AD) is the most common cause of dementia and characterized the accumulation of extracellular amyloid-β (Aβ) plaques and insoluble intracellular neurofibrillary tangles in the brain [1]. Aβ is the most important pathogenic factor for the development of AD, and causes several pathological changes, including memory loss, neuroinflammation, synaptic loss, and neuronal cell death [2]. In particular, Aβ-induced neuroinflammation is known to accelerate AD progression through neuronal damage induced by expression of inflammatory mediators [3,4]. Similarly, it has been known that lipopolysaccharides (LPS)-provoked neuroinflammation can also cause synaptic loss and induce cognitive impairment in AD [5,6]. In addition, LPS-mediated inflammation might be associated with enhancement of Aβ production [5,7,8]. Thus, both Aβ and LPS are one of the cause factors for the pathogenesis of AD by inducing neuroinflammation [5,9,10].

The microglia, which are the innate immune effector cells in central nervous system, have dual conflicting roles, such as neurotoxic and neuroprotective action, in the pathogenesis of neurodegenerative diseases, and these roles are associated with different functional phenotypes of microglia: M1 and M2 [11,12,13]. Switching of the microglial phenotype to M1 or M2 is regulated by pathological conditions. Toxins bind to the microglia cell surface and activate the M1 phenotype, which then produces neurotoxic cytokines [14,15]. Thus, persistent or unregulated neuroinflammation can cause tissue damage and secondary injury [16,17]. Conversely, M2 anti-inflammatory microglia promote tissue remodeling and repair by releasing anti-inflammatory cytokines [15,18]. Therefore, suppressing M1 microglial polarization which is subsequent release of pro-inflammatory molecules and/or enhancing the secretion of beneficial anti-inflammatory molecules from M2-polarized microglial cells could be a potential therapeutic approach for the treatment of AD [19,20].

Triggering receptor expressed on myeloid cells 2 (TREM2) is a type of membrane protein present on the surface of microglia and plays key roles in proliferation and survival of microglia, and secretion of inflammatory cytokines from microglia [21]. In the BV-2 microglial cell, knockdown of TREM2 induces the polarization to M1 phenotype, and overexpression of TREM2 promotes shifting to M2 phenotype of microglia [22]. Interestingly, TREM2 is known to be essential for microglia to recognize and phagocytose the Aβ in the brain with AD [23]. In addition, it has been known that TREM2 may be activated by various ligands, such as Aβ, LPS, apolipoproteins, and lipoproteins [24,25]. Moreover, TREM2 expression is decreased by pro-inflammatory stimuli, such as tumor necrosis factor alpha (TNF-α), interleukin-1 beta (IL-1β), or LPS [26,27,28]. Furthermore, knockdown of TREM2 in microglia suppresses phagocytic activity and activates gene transcription of TNF-α, whereas overexpression of TREM2 in microglia not only promotes phagocytosis but also diminishes pro-inflammatory responses [29]. Particularly, it was shown that up-regulated TREM2 expression in the 5XFAD mice reduced Aβ plaque and neurite dystrophy, and improved cognitive function and promoted microglial phagocytosis [30]. Furthermore, a number of studies have shown that TREM2 is related with microglial phenotype switching in neurological disorders, including AD [22,31,32,33,34]. Taken together, it can be suggested that microglia polarization might be associated with the TREM2 in the microglia within AD brain.

Although some of studies demonstrated that the low-dose ionizing radiation (LDIR) shows negative impacts on neurogenic precursor populations, dendritic spine density and cognitive dysfunction [35,36,37], several studies demonstrated that LDIR could be an effective intervention for the treatment of AD [38,39,40,41]. A number of reports have shown that LDIR significantly influences cognitive improvement and memory loss in AD [42,43,44,45]. In addition, it has been reported that ionizing radiation to the brain reduces deposition of Aβ plaque in Aβ-overexpressing transgenic models of AD [45]. Furthermore, LDIR decreases pro-inflammatory cytokines, such as interferon-gamma (INF-γ) and TNF-α, in animal models of AD [45,46]. However, there has been no studies about (1) the mechanisms how the radiation therapy reduces the Aβ accumulation and cognitive deficit in AD, and (2) the effect of LDIR therapy on M1/M2 polarization of microglia.

In this perspective, we hypothesized that LDIR-exposure would modulate the polarization of microglia phenotype and the expression of TREM2, resulting in amelioration of Aβ deposition and cognitive decline in AD. We found that LDIR affects microglial phenotype changes and the up-regulation of TREM2 in the LPS-treated microglia cell and Aβ-overexpressing mice. In this study, we demonstrated that LDIR causes the phenotype switching, which finally attenuated Aβ deposition and cognitive decline in AD.

## 2. Results

### 2.1. LDIR Inhibits Aβ Deposition and Improved Cognitive Deficits in 5XFAD Mice

**Figure 1:**
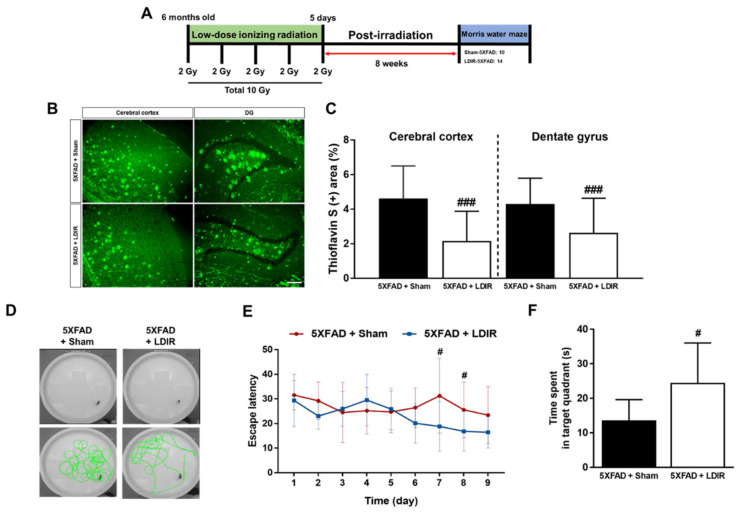
The inhibitory effects of low-dose ionizing radiation (LDIR) therapy on amyloid-β (Aβ) accumulation and cognitive dysfunction in 5XFAD mice. (**A**) Schematic diagram of the experimental procedure. (**B**) Histochemical staining for thioflavin S was performed to identify amyloid plaques in the cerebral cortex and hippocampal dentate gyrus (DG) of the sham- and LDIR-treated 5XFAD mice (scale bar = 250 μm). (**C**) Aβ-positive areas in the cerebral cortex and dentate gyrus of the sham- and LDIR-treated 5XFAD mice were quantified and plotted as a percentage. (**D**) Representative of swimming paths was recorded on videotape during the test session of the Morris water maze. (**E**) Latency to escape time of the mice was measured. Repeated-measures two-way ANOVA with Fisher’s post hoc test compared to 5XFAD+Sham mice. (**F**) Measurement of quadrant time to spend in the hidden platform. Data are presented as mean ± SD (*n* = 10 mice in sham-treated 5XFAD mice and *n* = 14 mice in LDIR-treated 5XFAD mice). ^#^ *p* < 0.05 and ^###^ *p* < 0.001 indicate significant differences between the sham- and LDIR-exposed 5XFAD mice.

It has been known that LDIR reduces the number of amyloid plaques and plaques size [43,45], and improves cognitive decline in the animal models of AD [44,45,47]. We reaffirmed the effect of the LDIR on Aβ deposition and cognitive function in the 5XFAD mice after exposure to 10 Gy radiation over 5 days (Figure 1A). We performed Morris water maze used in the previous studies, which demonstrated the alleviatory effect of LDIR on cognitive decline at 8 weeks after radiation therapy [44,45]. To confirm whether the LDIR inhibits Aβ aggregation in the cerebral cortex and hippocampus, brain sections were labeled by thioflavin S staining (Figure 1B). In comparison to sham-treated 5XFAD mice, LDIR-treated 5XFAD mice exhibited significant decrease in Aβ-positive area in both cerebral cortex and hippocampus (Figure 1C). To further reconfirm whether LDIR effectively rescues cognitive dysfunction in AD, Morris water maze test was performed with 5XFAD mice. The representative swimming paths of mice in each group indicated that the sham-treated AD transgenic model mice performed more unnecessary swimming (Figure 1D). The LDIR-treated 5XFAD mice showed increase in the quadrant time compared to sham-treated 5XFAD mice (Figure 1F). Particularly, two-way ANOVA repeated measures with multiple comparisons revealed a significant decrease in the escape latency on day 7 and 8 of trials in the LDIR-treated 5XFAD group compared with sham-treated 5XFAD group (Figure 1E). Consistent with previous reports, our results indicated that LDIR treatment ameliorates the Aβ accumulation and cognitive impairment in the Aβ-overexpressing transgenic mice.

### 2.2. LDIR Regulates Aβ-Induced Production of Inflammatory Cytokines in the 5XFAD Mice

**Figure 2:**
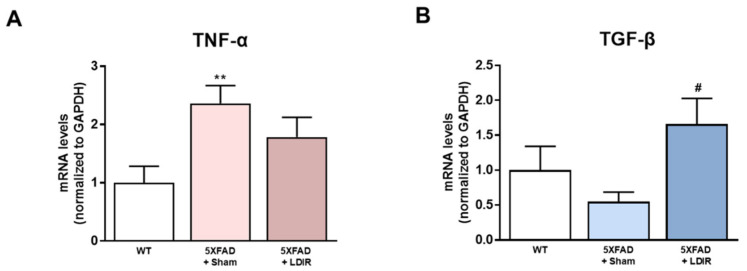
The modulatory effects of LDIR on production of M1/M2 cytokines in the brain of 5XFAD mice. (**A**) Level of TNF-α mRNA was checked by qRT-PCR. (**B**) Level of TGF-β mRNA was measured by qRT-PCR. ** *p* < 0.01 indicate significant differences between the wild-type (WT) and sham-treated 5XFAD mice. ^#^ *p* < 0.05 indicate significant differences between the sham- and LDIR-treated 5XFAD mice. Data are presented as mean ± SD (*n* = 3 mice each group).

It has been shown that LDIR exposure reduces the neuroinflammation in animal models of AD [41,45,46]. Inhibition of the M1 pro-inflammatory cytokines secreted from glial cells has been shown to attenuate synaptic dysfunction and enhance cognitive function in the AD [48]. In addition, activation of the M2 phenotype and inhibition of the M1 phenotype increased Aβ phagocytosis and clearance of amyloid plaques [49]. Therefore, we examined whether LDIR-induced improvement of cognitive function and reduction of Aβ deposition might be mediated by switching phenotype of microglial cells. To elucidate whether the LDIR may regulate microglial cytokine productions, representative cytokines of M1 and M2 microglia were examined in 5XFAD brain samples using qRT-PCR. We quantified mRNA levels of M1 pro-inflammatory marker TNF-α and M2 anti-inflammatory marker TGF-β in the brains of sham- and LDIR-treated 5XFAD mice. The levels of TNF-α mRNA were reduced in LDIR-treated 5XFAD compared to sham-treated 5XFAD mice (Figure 2A). In contrast, the levels of TGF-β mRNA were significantly increased in LDIR-treated 5XFAD compared to sham-treated 5XFAD mice (Figure 2B). Our results suggest that the M2 cytokine was up-regulated, and the M1 cytokine was suppressed after LDIR therapy in the brain of 5XFAD mice.

### 2.3. LDIR Modulates the Levels of M1/M2 Cytokines in LPS-Treated BV-2 Cells

**Figure 3:**
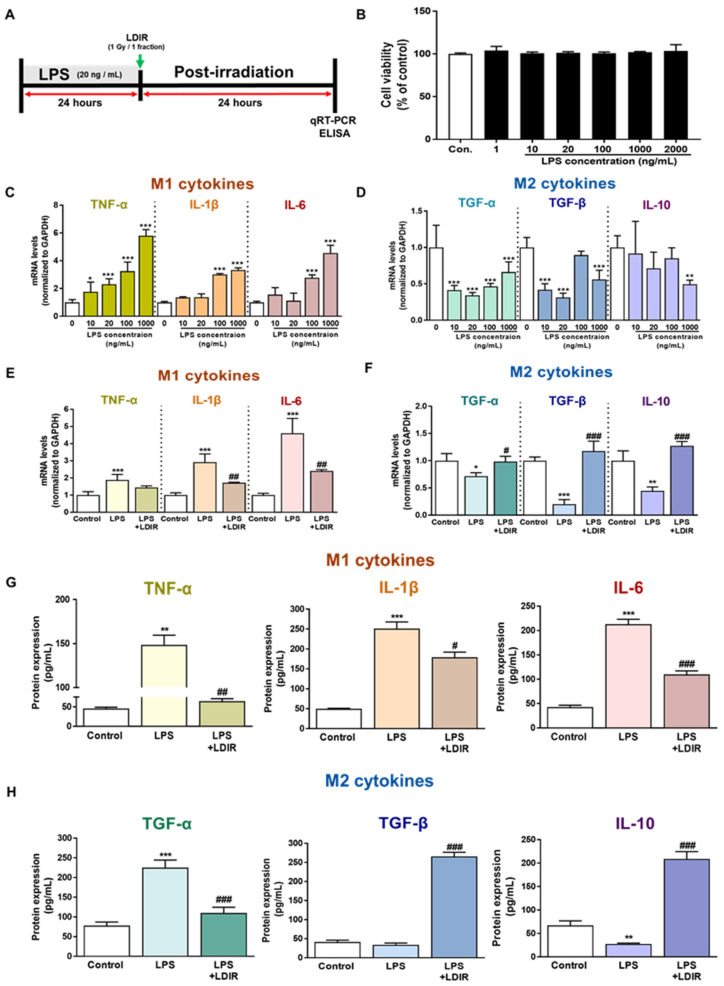
The modulatory effects of LDIR on production and secretion of M1 cytokines (TNF-α, IL-1β and IL-6) and M2 cytokines (TGF-α, TGF-β and IL-10) in lipopolysaccharide (LPS)-treated BV-2 cells. (**A**) Outline of the experimental design for in vitro assessment. (**B**) BV-2 cells were treated with LPS at doses of 1, 10, 20, 100, 1000 and 2000 ng/mL for 24 h. Cell viability was determined by the cell counting Kit-8 assay. (**C**,**D**) After BV-2 cells were treated with LPS (10, 20, 100, and 1000 ng/mL) for 24 h, the mRNA expression levels of M1 cytokines and M2 cytokines were measured by qRT-PCR. (**E**–**H**) BV-2 cells were treated with LPS (20 ng/mL) for 24 h, then exposed to LDIR (1 Gy/ 1 fraction). The mRNA expression levels of (**E**) M1 cytokines and (**F**) M2 cytokines were measured by qRT-PCR. The protein levels of (**G**) M1 cytokines and (**H**) M2 cytokines in cultured media were measured by ELISA. * *p* < 0.05, ** *p* < 0.01, and *** *p* < 0.001 indicate significant differences between the control and LPS-treated group. ^#^ *p* < 0.05, ^##^ *p* < 0.01, and ^###^ *p* < 0.001 indicate significant differences between the LPS- and LPS + LDIR- treated group. Data are presented as mean ± SD from triplicate experiments.

To confirm the direct effect of the LDIR on microglia polarization with M1/M2 phenotype in the AD brain, we selected the BV-2 cell, which is an immortalized neonatal mouse microglial cell line. First, to identify the cytotoxicity of LPS, the BV-2 cells were incubated with LPS at concentrations of 1, 10, 20, 100, 1000, and 2000 ng/mL for 24 h (Figure 3B). The concentrations of LPS and culture conditions tested were based on previous studies [50,51,52]. We aimed to determine the optimal concentration of LPS, which changed the mRNA levels of M1 and M2 cytokines in microglia but was the least toxic to these cells (Figure 3B–D). As depicted in Figure 3B, treatment with LPS for 24 h was not toxic to the cells. In addition, we also observed that various doses of LDIR exposure did not cause the toxicity of BV-2 cells (Figure S1). As shown in Figure 3C and Figure 3D, there was a significant change in the mRNA levels of M1 and M2 cytokines after LPS treatment. An amount of 20 ng/mL was chosen as a standard concentration for further experiments. The BV-2 cells were treated with LPS at a dose of 20 ng/mL and exposed to 1 Gy radiation for 1 fraction (Figure 3A). Expression levels of M1 cytokines (TNF-α, IL-1β and IL-6) and M2 cytokines (TGF-α, TGF-β and IL-10) were measured by qRT-PCR to assess the phenotype switching in BV-2 cells. After 24 h of LPS treatment, mRNA levels of M1 cytokines were markedly elevated (Figure 3E), while mRNA levels for M2 cytokines were dramatically reduced when compared to the control group (Figure 3F). However, LDIR treatment significantly reduced mRNA levels of M1 cytokines (Figure 3E) and elevated the mRNA levels of M2 cytokines (Figure 3F), indicating that the LPS-treated BV-2 cells revealed phenotype switching from M1 to M2 following LDIR treatment. Subsequently, in addition to mRNA levels of M1/M2 cytokines, we also measured the released M1/M2 cytokines from LPS-treated BV-2 cells without or with LDIR using ELISA. Secretion of M1 pro-inflammatory cytokines, such as TNF-α, IL-1 β, and IL-6, into the culture medium of BV-2 cells dramatically increased in response to LPS stimulation (Figure 3G). However, after LDIR, the release of M1 cytokines was significantly inhibited in LPS-stimulated microglia (Figure 3G). In addition, the production of M2 cytokines, such as TFG-β and IL-10, from LPS-treated BV-2 cells were significantly induced 24 h after LDIR (Figure 3H). Taken together, these data suggested that LDIR could effectively shift the LPS-induced M1 phenotype to an M2 phenotype of microglial cells.

### 2.4. LDIR Affects Changes of M1/M2 Phenotypes of LPS-Treated BV-2 Cells

**Figure 4:**
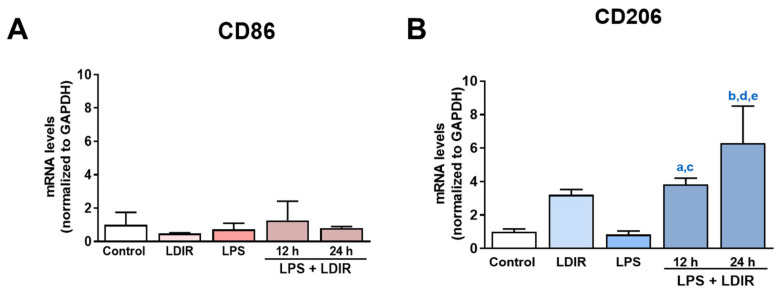
The effects of LDIR exposure on the expressions of M1/M2 markers in BV-2-stimulated cells. BV-2 cells were treated with LPS at a dose of 20 ng/mL for 24 h then exposed to LDIR (1 Gy/ 1 fraction). Cells were harvested at 12 and 24 h after irradiation. (**A**,**B**) The mRNA levels of M1 microglial marker CD86 and M2 microglial marker CD206 were measured by qRT-PCR. a indicated that comparison of control and LPS+LDIR 12 h (*p* < 0.05), b indicated that comparison of control and LPS+LDIR 24 h (*p* < 0.001), c indicated that comparison of LPS and LPS+LDIR 12 h (*p* < 0.05), d indicated that comparison of LPS and LPS+LDIR 24 h (*p* < 0.001), and e indicated that comparison of LDIR and LPS+LDIR 24 h (*p* < 0.05). Data are presented as mean ± SD from triplicate experiments.

Using the markers for M1 and M2 microglial phenotype [53,54,55], we tried to clearly demonstrate that LDIR can change the polarization of M1/M2 activation of LPS-treated microglial cells. We examined the expression of BV-2 cell surface receptors: M1 phenotypic marker CD86 and M2 phenotypic marker CD206. At the mRNA levels, the LPS or LDIR alone did not induce the inflammatory response on BV-2 cells compared with control group. In addition, there were no changes of CD86 in the LPS-treated BV-2 cells 12 and 24 h after LDIR exposure (Figure 4A). On the other hand, there were significant up-regulation of CD206 in the LPS-stimulated BV-2 cells 12 and 24 h after LDIR exposure (Figure 4B). We demonstrated that M2 polarization of microglia can be induced by LPS+LDIR treatment.

### 2.5. LDIR Induces the Up-Regulation of TREM2 in LPS-Treated BV-2 Cells

**Figure 5:**
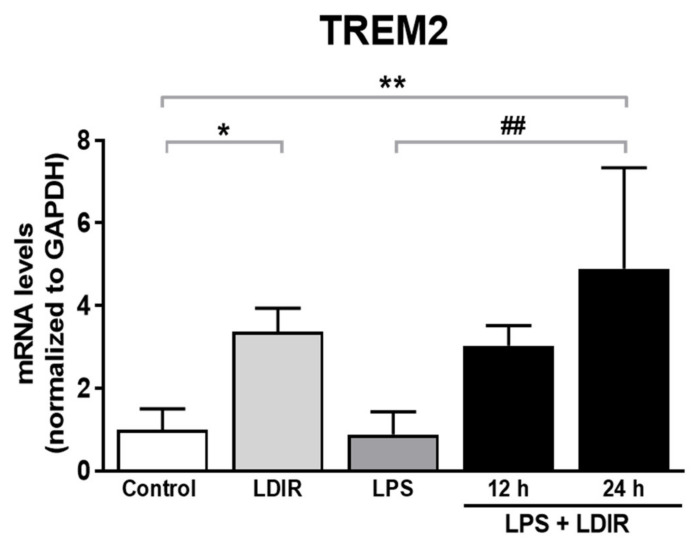
The enhancing effects of LDIR exposure on expression of TREM2 in LPS-stimulated BV-2 cells. BV-2 cells were treated with LPS at a dose of 20 ng/mL for 24 h, and then were exposed to LDIR (1 Gy/ 1 fraction). Cells were harvested at 12 and 24 h after irradiation. After irradiation and/or LPS treatment, levels of TREM2 mRNA were detected by qRT-PCR in BV-2 cells. * *p* < 0.05, ** *p* < 0.01 indicate significant differences to control group. ^##^ *p* < 0.01 indicate significant differences to LPS group. Data are presented as mean ± SD from triplicate experiments.

TREM2, one of microglial transmembrane receptors, is known as a regulator of the microglial M1/M2 polarization and microglial responsivity in AD [22,30]. We examined whether the LDIR may affect the expression of TREM2 in microglia showing polarization to M2 phenotype. We found the LDIR-mediated up-regulation of TREM2 mRNA level in BV-2 cells, compared with control groups. Furthermore, LPS-treated BV-2 cells exposed to LDIR showed significant up-regulation of TREM2 mRNA in BV-2 cells, compared with LPS-treated groups (Figure 5). Taken together, our data suggest that LDIR treatment could simultaneously induce M2 polarization of microglia and the upregulation of TREM2 in activated microglia.

## 3. Discussion

**Figure 6:**
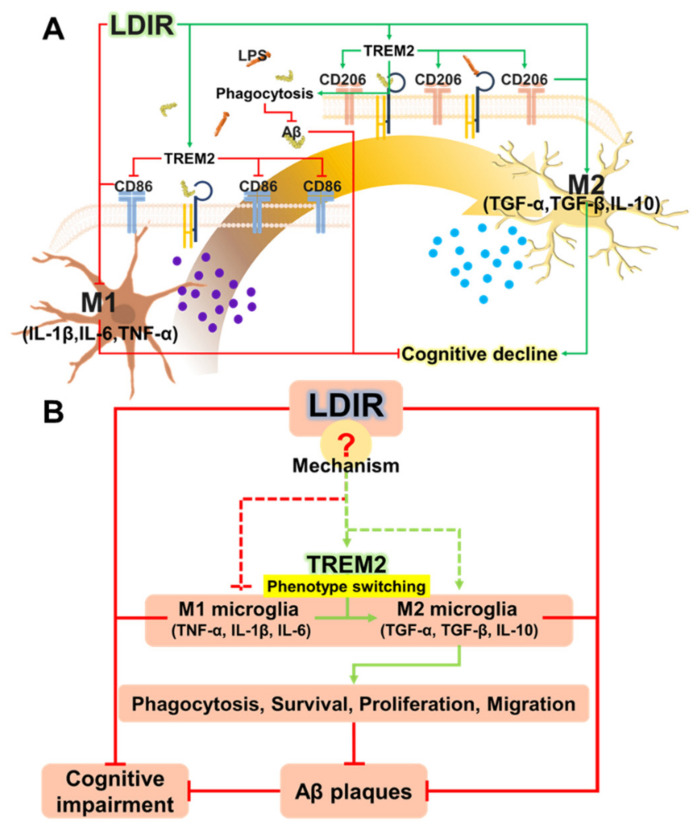
(**A**) Schematic drawing of the modulatory effects of LDIR exposure on shifting microglial phenotypes. (**B**) Proposed mechanisms for how LDIR treatment is able to reduce Aβ accumulation and cognitive decline.

Recently, studies about LDIR exposure for the treatment of AD have been increasing [38,39]. Although previous studies have reported the beneficial effect of the LDIR therapy on the Aβ pathology and cognitive decline in AD [43,45], there has been few studies about the mechanism of the LDIR therapy for the treatment of AD. Therefore, we examined the effects of the LDIR on microglial phenotypes and expression of TREM2 in AD models. Consistent with previous reports, our results also revealed that LDIR inhibits Aβ deposition and cognitive dysfunction in 5XFAD mice (Figure 1). Intriguingly, exposure to LDIR regulated the expression of pro-inflammatory and anti-inflammatory cytokines in the brain of Aβ-overexpressing transgenic mice (Figure 2). Next, we examined the direct effect of LDIR on the changes of M1/M2 cytokines in cultured microglial BV-2 cells. Surprisingly, the mRNA and protein levels of M1 proinflammatory cytokines, such as TNF-α, IL-1β, and IL-6, were significantly reduced and the mRNA and protein levels of M2 anti-inflammatory cytokines, such as TGF-α, TGF-β, and IL-10, were significantly increased in the LPS-treated microglia after the exposure of LDIR when compared to LPS-stimulated cells (Figure 3). Moreover, the LDIR treatment significantly increased expression of M2 phenotypic marker CD206 in LPS-treated microglia (Figure 4), suggesting that the interaction between LPS and LDIR therapy can switch the microglial phenotype from M1 to M2. Additionally, expression of TREM2 involved in microglial M1/M2 polarization was increased after the LDIR exposure in LPS-stimulated microglia (Figure 5). Taken together, it has been suggested that the LDIR treatment inhibits the cognitive deficit and Aβ deposition in AD by modulating the polarization of microglial phenotype and expression of TREM2 (Figure 6A).

Plasma levels of LPS in AD patients have been reported to be three times higher than in normal individuals [56]. Similar to Aβ, LPS is known to cause inflammation in the central nervous system [57]. LPS significantly contributes to the pathogenesis of AD and is well-established as a one of the risk factors for AD [5,9,10]. In addition, LPS-induced inflammation acts as an upstream of Aβ pathology by increasing Aβ production [5,7,8]. Moreover, LPS damages the blood brain barrier (BBB), allowing more LPS to enter the brain [58]. Furthermore, LPS interacts directly with microglia to induce acute inflammatory responses, and neurotoxic molecules from activated microglia are well known to cause the neurodegeneration [57,59,60,61]. Our LPS-treated results are inconsistent with previous studies showing that LPS-induced microglial phenotypic changes characterized by increase of M1 marker, and decrease of M2 marker [62,63,64,65]. Since various factors such as cell line, serotype, species, dose of LPS, and batch of LPS contents have affected the inflammatory reaction, we speculated that our results were not consistent with the previous studies. Although CD 86 and CD 206 were not significantly changed in this study (Figure 4), it is certain that LPS provoked an inflammatory response and affected the microglial phenotype changes. Notably, LPS has been reported to downregulate TREM2 expression through the NF-κB pathway [66,67]. Surprisingly, in our study, LPS+LDIR or LDIR-induced upregulation of TREM2 was detected at the level of mRNA, and is related with inhibition of pro-inflammatory cytokines and consequently suppression of AD pathology [68]. Taken together, not only Aβ but also LPS can be identified as a critical factor that disrupts microglial phenotype-related homeostasis in AD [32]. Therefore, LPS is one of the inflammatory triggers in AD progression, indicating that LPS-mediated inflammation can directly contribute to pathogenesis of AD [69].

In AD, it has been shown that Aβ and tau result in a hyper-activated M1 phenotype of microglial cells, leading to detrimental brain damage concomitant with the expression of pro-inflammatory cytokines, such as TNF-α, IL-1β, and IL-6. In contrast, the M2 phenotype of microglial cells can induce neuroprotective functions and restore the brain environment through the action of neurotrophic factors [70]. In addition, it has been known that, after exposure to LDIR, air-pouch mouse show the down-regulation of TNF-α and IL-1β, which are the representative M1 cytokines, whereas the expression of anti-inflammatory M2 cytokine TGFβ-1 was up-regulated in air-pouch mouse [71]. In the present study, we examined whether the phenotype of microglia cells could be changed by LDIR therapy through the detection of M1 and M2 cytokines in in vivo and in vitro models of AD. We found that the LDIR treatment down-regulated of M1 cytokines and up-regulated M2 cytokines in 5XFAD mice or BV-2 cells activated by LPS (Figure 2 and Figure 3). In order to confirm the phenotype change of microglia cells specifically, further study is needed to perform the double labeling of microglia cells and TNF-α and TGF-β. Taken together, these data indicate that LDIR therapy strongly modulates the release of M1 and M2 cytokines in the brain with AD.

TREM2 has been known to be a major regulator for microglial phenotype switching [32]. Heterozygous rare variants (R47H) of TREM2 in AD reduce the expression of TREM2 and cell surface transport function [24,72]. TREM2 deficiency in the AD animal models resulted in reduction of the number of microglia around the Aβ plaques and aggravation of the Aβ pathology [73,74,75]. In addition, microglia with decreased TREM2 levels predominantly exhibited the M1 phenotype and secreted neurotoxic cytokines [76]. In contrast, the secretion of pro-inflammatory cytokines by M1 polarization was reduced in TREM2-overexpressing mouse model [31]. Furthermore, increased expression of TREM2 resulted in both reduction of M1 microglia, which contribute to AD pathogenesis by inducing pro-inflammatory responses and neuronal damage [77], and increase of M2 microglia, which ameliorate AD pathology through release of anti-inflammatory cytokines and neurotropic factors [78]. In the BV-2 cell, knockdown of TREM2 expression increases M1 inflammatory responses and inhibits phenotype switching to M2, while overexpression of TREM2 promotes phenotype change to M2 and alleviated inflammation of M1 [22]. Interestingly, our results revealed that LDIR increased mRNA levels of TREM2 in LPS-treated BV-2 cells (Figure 5). Accordingly, it seems likely that the LDIR therapy might induce M1/M2 phenotype changes via increasing the expression of TREM2 in the microglia within AD brain (Figure 6B). However, it should be cautioned to interpret our results because the microglia polarization concept is controversial in the neuroscience field.

Radiation therapy (20 Gy/10 fractions) has been used for decades to treat extra-cranial amyloidosis, especially when the deposits cause airway obstructions in the trachea [79,80]. Recent studies have emerged that address the potential application of LDIR in treating AD caused by accumulation of misfolded proteins such as Aβ and tau [79]. Current therapies for AD only target specific proteins, such as Aβ or tau, and these clinical studies targeting for Aβ or tau have failed to improve the cognitive function of patients. However, LDIR not only targets pathogenic protein, such as Aβ, but also has an indirect effect that involves the phenotype shifting microglial cells. The data presented in this study suggest that LDIR therapy could effectively improve cognitive function in AD.

Although the mechanism underlying the effects of radiation therapy for AD has not yet been completely elucidated, previous reports and the results of the current study have suggested the possible therapeutic mechanisms mediated by LDIR exposure. Firstly, LDIR may cause neuroprotection via up-regulation of neurotrophic factors, such as TGF-β and IL-10, as reported in the present study. In addition, TGF-β has also been reported to be involved in cell growth and maintenance [81,82]. Additionally, we discovered that LDIR provokes microglia phenotype shifting from an M1 to M2 phenotype under LPS and AD treatment. Thus, increased phagocytosis mediated by M2 polarization can lead to an increase in clearance of abnormal proteins such as Aβ [83]. Secondly, LDIR up-regulates the expression of synaptophysin and intercellular adhesion molecules (CAM) [84,85], which enable synaptic plasticity and promote synaptogenesis. Enhancement of synaptogenesis can improve cognitive function [86]. Thirdly, LDIR up-regulates heat shock protein 70, a protein that has been reported to regulate misfolded protein aggregation and may reduce the toxicity of Aβ [87,88]. Finally, LDIR induces up-regulation of vascular endothelial growth factors (VEGF), which enhances the patency of the dysfunctional lymphatic pathway triggered by AD pathology and is subsequently involved in the drainage of Aβ [89,90,91,92,93].

Based on the abovementioned mechanisms, in August 2019, our research team initiated a study investigating the effect of 2 regimens of LDIR therapy (9 Gy/5 fraction and 5.4 Gy/3 fraction) on Aβ plaque formation and cognitive function in patients with early or moderate AD. We started a randomized prospective phase II clinical trial to assess if LDIR therapy could inhibit cognitive decline in AD (NCT 04203121). Currently, approximately six clinical trials have been conducted, not only in South Korea, but also in the US and Europe and are expected to yield effective clinical outcomes within a few years.

## 4. Materials and Methods

### 4.1. Reagents

LPS from *Escherichia coli*, serotype 026: B6, was purchased from the Sigma-Aldrich Chemical Company (St. Louis, MO, USA). Thioflavin S was purchased from Sigma-Aldrich Chemical Company (T1892-25G).

### 4.2. Animals

The male heterozygous 5XFAD transgenic mice (C57BL/6-SJL background; 6-month-old; obtained from Jun biotech, Inc, Republic of Korea) were used. The 5XFAD mice was identified by genotyping. Wild-type (WT) littermates of 5XFAD mice were used controls. Mice were sacrificed 8 weeks after LDIR treatment. All experiments detailed herein complied with the regulations specified by Kyung Hee University Guidelines for Laboratory Animal Care and Use. This animal research was approved by committee of Kyung Hee University Hospital in Gangdong (project identification code: KHNMC AP 2017-003, date: 21 February 2017).

### 4.3. Radiation Exposure

X-ray irradiating system (LEP-300, Auracare^®^, Gyeonggi-do, Korea) and X-ray tube (320 kVp, 15 mA, Varian Medical Systems, Inc., USA) were used for all irradiation procedures, which were performed at room temperature. For the in vivo experiments, mice were anesthetized with 2.5 mg/kg Zoletil, placed in an immobilizer, and exposed to a total radiation dose of 10 Gy/5 fractions. Cells were cultured in a 60 × 15 mm dish. Immediately before irradiation exposure, the culture dish was filled with media and all air gaps were eliminated. The cells were then exposed to a 50, 100, 200, 380, or 525 cGy/fraction of radiation (Figure S1). Lead shields protected the sham-treated cells and mice during exposure of the radiation.

### 4.4. Memory Test

The spatial learning and memory of mice were evaluated using the Morris water maze task, as previously described [94]. The experimental procedures were recorded on videotape.

### 4.5. Confocal Microscopy

Brain tissue blocks were cryo-sectioned at a thickness of 7 μm. For Aβ histology staining, the sections were incubated in 0.5% thioflavin-S (50% in ethanol) for 10 min, then washed with 50% ethanol, and finally with PBS. Fluorescence signal was assessed using the Zeiss LSM 700 laser confocal microscopy system.

### 4.6. Real-Time Quantitative Reverse Transcription Polymerase Chain Reaction

Total RNA was extracted from BV-2 cells or brain samples of mice using an RNA purification kit (Gene All, Seoul, Republic of Korea) according to the manufacturer’s protocols. Complementary DNA (cDNA) was generated using Revert Aid First Strand cDNA Synthesis Kit (Thermo Scientific, Waltham, MA, USA) and was subjected to qRT-PCR analysis using SYBR Green PCR master mix (Thermo Scientific, Waltham, MA, USA). The gene-specific primer sequences for M1/M2 markers and GAPDH are listed in Table S1. The relative expression of each gene was calculated using the ΔΔCq method with GAPDH. For quantification, the quantification cycle (Cq) values were normalized with GAPDH Cq and analyzed with the Comparative CT Method 2^−ΔΔCq^ method, as previously described [95].

### 4.7. Enzyme-Linked Immunosorbent Assay

The effect of LDIR exposure on the protein levels of M1 (TNF-α, IL-1β, and IL-6) and M2 (TGF-α, TGF-β, and IL-10) cytokines was analyzed by ELISA. Cell-free culture supernatants were collected and 50 μL of undiluted cultured media per well was analyzed for M1 or M2 cytokines by a specific ELISA kit (mouse inflammatory cytokine multianalyte ELISA array kit; Qiagen) according to the manufacturer’s recommendations.

### 4.8. BV-2 Cell Culture

The murine microglia cell line BV-2 were maintained at 37 °C and 5% CO_2_ in Dulbecco’s modified Eagle’s medium (DMEM) supplemented with 2 mM glutamine, 100 μg/mL streptomycin, 100 units/mL penicillin, and 10% heat-inactivated fetal bovine serum (FBS).

### 4.9. Cell Viability

The Cell counting kit-8 (CCK-8) assay was used to determining of cell viability after treatment of LPS and LDIR. CCK-8 assay was conducted following the manufacturer’s protocol (Dojindo Molecular Technologies, Kumamoto, Japan). Emax Plus Microplate Reader (Molecular Devices Emax, CA, USA) was used to measure the absorbance of each well at 540 nm.

### 4.10. Immunoblotting

The BV-2 cells were homogenized with RIPA buffer and protease inhibitors (Thermo Scientific). Equal amounts of protein (40 μg/lane) were loaded to gradient (4 to 15%) concentrations of polyacrylamide SDS-PAGE (Smobio, Hsinchu City, Taiwan) and transferred to polyvinylidene difluoride (PVDF) membranes (Millipore, Billerica, MA) using an electro-transfer system (Bio-Rad, Hercules, CA). The membranes were blocked with 5% nonfat milk and probed with the following primary antibodies for 16 h at 4 °C: rabbit polyclonal anti-CD206 (1:1,000; Abbkine, Wuhan, China), goat polyclonal TREM2 (1:1,000, ThermoFisher Scientific), and mouse monoclonal β-actin (1:5,000; Abbkine). The membranes were incubated with the appropriate secondary antibodies (Bethyl, Inc., Montgomery, TX, USA) conjugated to horseradish peroxidase (HRP) for 1 h at room temperature. Bound antibodies were visualized with D-Plus ECL Pico System (Dongin LS, Republic of Korea) and a G: Box Chemiluminescence & Fluorescence system (Syngene, Frederick, MD, USA).

### 4.11. Statistics

Data are presented as means ± SD. Paired samples’ statistics using the student’s *t*-test was conducted for analysis between sham-exposed and LDIR-exposed groups. For escape latency, repeated measure two-way analysis of variance (ANOVA) was used. The other analysis performed using the one-way ANOVA followed by Fisher’s LSD post-hoc test for comparisons among the three groups. Asterisks indicate significant differences, as noted in the figure legends.

## 5. Conclusions

For the first time, we studied the effects of LDIR on polarization of microglia using both in vitro and in vivo models. LDIR not only affected the microglia phenotype shifting, but also clears amyloid plaques, resulting in inhibition of cognitive decline in AD. In future studies, we aim to focus on elucidating the mechanism by which LDIR affects these changes (Figure 6B), as well as identifying the optimal LDIR treatment regimen for diverse neurodegenerative diseases with a specific focus on AD.

## Supplementary Materials

Supplementary materials can be found at https://www.mdpi.com/1422-0067/21/12/4532/s1.

## Abbreviations

AD	Alzheimer’s disease
ANOVA	one-way analysis of variance
Aβ	amyloid-β
BBB	blood brain barrier
CAM	intercellular adhesion molecule
CCK-8	Cell counting kit-8
DMEM	Dulbecco’s Modified Eagle Medium
IL-1β	interleukin-1 beta
INF-γ	interferon-gamma
LDIR	Low-dose ionizing radiation
LPS	lipopolysaccharides
R47H	Heterozygous rare variants
TNF-α	tumor necrosis factor alpha
TREM2	triggering receptor expressed on myeloid cells 2
VEGF	vascular endothelial growth factors
WT	Wild-type
