# Supplementary material for: Low-Dose Ionizing Radiation Modulates Microglia Phenotypes in the Models of Alzheimer’s Disease
Source: Int J Mol Sci. 2020 Jun 25;21(12):4532. doi: 10.3390/ijms21124532 (PMC7353052; doi:10.3390/ijms21124532)
Supplement: Supplementary file 1 [file ijms-21-04532-s001.pdf]

Supplementary figure

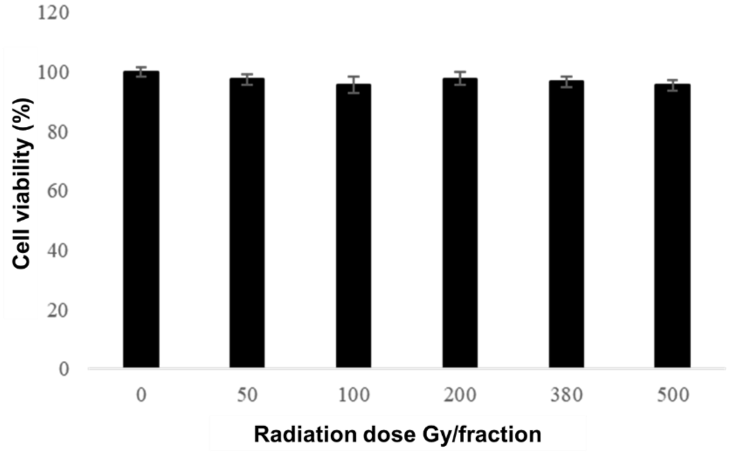

Supplement figure 1. **BV-2 cells were exposed to various dose of LDIR.** 12 h after treatment, cell proliferation was determined by Cell counting Kit-8. Data are presented as mean ± SEM from triplicate experiments.

Primer		Sequence
**TNF-α**	Sense	5’- TCGTAGCAAACCACCAAGTG-3’
	Antisense	5’- ATATAGCAAATCGGCTGACG -3’
**IL-6**	Sense	5'-GAGGATACCACTCCCAACAGACC-3'
	Antisense	5'-AAGTGCATCATCGTTGTTCATACA-3'
**IL-1β**	Sense	5’-CCTTCCAGGATGAGGACATGA -3’
	Antisense	5’-TGAGTCACAGAGGATGGGCTC -3’
**TGF-α**	Sense	5’-TACCGCTGGGTATCCTGTTA-3’
	Antisense	5’-TTCTCATGTCTGCAGACGAG-3’
**TGF-β**	Sense	5′-AAGAAGTCACCCGCGTGCTA-3′
	Antisense	5′-TGTGTGATGTCTTTGGTTTTGTCA-3′
**IL-10**	Sense	5’-CCAAGCCTTATCGGAAATGA-3’
	Antisense	5’-TGGCCTTGTAGACACCTTGG-3’
**TREM-2**	Sense	5’-GACCTCTCCACCAGTTTCTCC-3’
	Antisense	5’-TCAGAGTGATGGTGACGGTTC-3’
**GAPDH**	Sense	5′- TGGCACAGTCAAGGCTGAGA-3′
	Antisense	5′-CTTCTGAGTGGCAGTGATGG-3′

Supplement table 1. primer sequences used for qRT-PCR.
